# Characterization of amputees at a large hospital in Recife, PE, Brazil

**DOI:** 10.1590/1677-5449.190064

**Published:** 2019-10-23

**Authors:** Ylkiany Pereira de Souza, Ana Célia Oliveira dos Santos, Luciana Camelo de Albuquerque

**Affiliations:** 1 Universidade Federal de Pernambuco – UFPE, Programa de Pós-graduação em Gerontologia, Recife, PE, Brasil.; 2 Universidade de Pernambuco – UPE, Instituto de Ciências Biológicas – ICB, Recife, PE, Brasil.; 3 Prefeitura da Cidade de Recife, Distrito Sanitário 2, Recife, PE, Brasil.

**Keywords:** amputation, disarticulation, upper limbs, lower limbs

## Abstract

**Background:**

Limb amputation can be defined as a procedure that consists of separating a limb or a segment of a limb from the body.

**Objectives:**

To describe the profile of limb amputation procedures performed at a large hospital run by the state of Pernambuco (Brazil).

**Methods:**

Cross-sectional descriptive and retrospective study conducted at a large hospital in the city of Recife, PE. Data were collected from the records of patients who underwent amputations during 2017. Records from patients who had had a limb amputation during 2017 were included, unless data were illegible or missing.

**Results:**

A total of 328 procedures were performed on 274 patients, the majority of whom were male (57.7%). There was a predominance of lower limb amputations (64.2%), of non-traumatic causes (86.5%), and urgent treatment (96.4%). The majority of patients who underwent amputations remained in hospital for 11 to 25 days (32.1%). The study found that the majority of amputees were discharged (69.7%), although a proportion died. Deaths of lower limb amputees were primarily among elderly women in the age range of 60 to 90 years (76%), females (55%), and patients subjected to a single amputation (91%).

**Conclusions:**

The data observed in this study are alarming, particularly considering that many of these amputations could have been avoided, since they were caused by complications of diseases that can be prevented and controlled at healthcare services of a lower level of complexity and at a relatively low cost.

## INTRODUCTION

Limb amputation is a major public health problem, responsible for high rates of morbidity and mortality, and the loss of a limb or part of a limb impacts on a person’s psychosocial status and also increases costs for health services. Amputations are generally performed as a last resort in an attempt to reinstate a person’s health, when all other options have already been employed or are unfeasible, whether because of irreversible ischemia or trauma with excessive destruction of the limb involved.^1^^-^ 3

Causes that lead to amputation include infectious and parasitic diseases, diseases of the circulatory apparatus, diabetes mellitus (DM), gangrene, diseases of the musculoskeletal system and the connective tissues, cancer, external causes, skin diseases, and congenital malformations.^2^ Amputation procedures may therefore by urgent operations or may be scheduled electively.^1^

According to the few studies investigating the epidemiology of amputations, it is estimated that the global incidence of limb amputations is approximately 1 million people per year.^4^^,^ 5 In 2018, over 59,000 amputations were recorded in Brazil, 2,694 of which were performed in Pernambuco state, putting the state in eighth place in terms of number of amputations.^6^

Lower limb (LL) amputations are generally more common than upper limb (UL) amputations. According to data from the Brazilian Ministry of Health (MS), in 2011, LL amputations accounted for around 94% of all amputations performed.^2^

The objective of this study was to describe the profile of limb amputation procedures performed at a large hospital run by the state of Pernambuco. The results can be used to support healthcare managers when taking decisions on public policies that could reduce the number of amputations.

## METHOD

This is a retrospective, descriptive, cross-sectional study conducted at a large hospital located in the city of Recife, PE, with the objective of analyzing hospital data on patients who underwent limb amputations from January 2017 to December 2017.

Data collection was conducted from May to September 2018 by a single investigator, who completed a form containing the following variables: sex, age, type of amputation, procedure employed, type of care (emergency or elective), number of amputations, cause of amputation, origin, reason for exit from hospital, and length of hospital stay.

In order to confirm the number of amputations performed and to obtain information on all of the variables covered in the instrument, the investigator completed the form using three different sources of data: 1) records maintained at the surgical center, using the center’s surgery registers; 2) mortuary referral documents; and 3) copies of hospital admission authorizations (HAA) available from the accounts department as part of a data input support service (SISAIH01).

It should be pointed out that since the SISAIH01 system was used to obtain copies of the HAA records, it was not possible to precisely determine the level of amputation performed, i.e., whether transtibial, transfemoral, etc., because this system combines several different levels under a single label. For instance, the two examples mentioned are both part of an LL amputation/disarticulation category.

**Figure 1:**
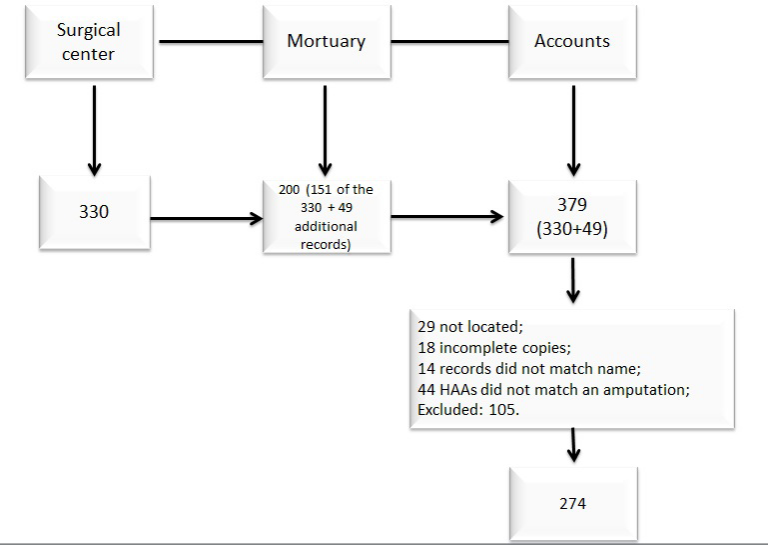
Flow diagram of data collection stages, showing number of records identified, selected, and included in the study. HAA = hospital admission authorizations.

At the surgical center, 330 records of people subjected to limb amputations were acquired and 200 records were found at the mortuary. At that point, it was found that many of the surgical center records were not among those held at the mortuary, and vice-versa, although all amputated limbs are sent to the mortuary with the referral document. In summary, 151 of the 330 records acquired from the surgical center coincided with the data from the mortuary, and an additional 49 records were found at that mortuary. The 330 records from the surgical center and the additional 49 from the mortuary that were not held at the surgical center were checked at the accounts department, a total of 379 records to investigate. However, some of these were excluded during analysis of the HAA data, because of the following reasons: incomplete HAAs, records that did not coincide with the name, HAAs that did not match an amputation, repeated records, and missing records. The final number of records collected was 274 (Figure 1), with 105 losses.

The data collected were input to Microsoft Excel® for descriptive analysis of the data, computing absolute frequencies (n) and proportions (%) for categorical/qualitative variables of interest and means (± standard deviations) for quantitative variables. The study complied with all ethical considerations for research with human beings, in accordance with Resolution 466/2012^7^ and was submitted to a Research Ethics Committee, using the Plataforma Brasil, and was approved under decision number 2.622.379.

## RESULTS

A total of 328 amputation procedures were performed on 274 patients with ages ranging from 13 years to > 90 years, with a mean of 63 years (standard deviation ±16). The age group with the highest frequency of amputees was 60 to 90 years (60.2%; n =165), and a majority of the amputees (57.7%; n = 158) were male (Table 1).

**Table 1:** Distribution of patients according to sociodemographic study variables. Recife, PE, Brazil, 2019.

**General characteristics**	**n**	**%**
Age (mean ± standard deviation), n = 274	63±16	
13 to 59 years	100	36.5
60 to 90 years	165	60.2
Over 90 years	9	3.3
Sex, n = 274		
Male	158	57.7
Female	116	42.3

A majority of amputations were non-traumatic, accounting for 86.5% (n = 237). Analysis of the number of amputations per patient showed that the great majority of patients (83.2%; n = 228) underwent a single amputation. With regard to the procedure performed, LL amputation/disarticulation was the most prevalent (53.6%; n = 176), followed by amputation/disarticulation of fingers or toes (23.4%; n = 77) (Table 2).

**Table 2:** Distribution of patients according to the characteristics of amputations. Recife, PE, Brazil, 2019.

**General characteristics**	**n**	**%**
Type of amputation, n = 274		
Traumatic	37	13.5
Non-traumatic	237	86.5
Number of amputations, n = 274		
One	228	83.2
Two	38	13.8
Three	8	2.9
Amputation procedure, n = 328		
Amputation/Disarticulation of UL	4	1.2
Amputation/Disarticulation at hand or wrist	2	0.6
Amputation/Disarticulation of LL	176	53.6
Amputation/Disarticulation at foot or ankle	69	21.0
Amputation/Disarticulation of fingers or toes	77	23.4
Cause of amputation, n = 305		
I Certain infectious and parasitic diseases	244	80
II Neoplasms [tumors]	2	0.6
IV Endocrine, nutritional and metabolic diseases	2	0.6
IX Diseases of the circulatory system	32	10.4
XII Diseases of the skin and subcutaneous tissue	2	0.6
XIII Diseases of the musculoskeletal system and connective tissue	1	0.3
XVIII Symptoms, signs and abnormal clinical and laboratory findings, not elsewhere classified	13	4.2
XIX Injury, poisoning and certain other consequences of external causes	8	2.6
XXI Factors influencing health status and contact with health services	1	0.3

UL: upper limbs; LL: lower limbs.

With regard to the distribution of major amputations (those performed at more proximal levels) and minor amputations (at more distal levels), it was found that 54.8% (n = 180) of the amputations were major amputations, which were those coded as LL amputations, signifying proximal lower limb amputations, and those coded as UL, signifying upper limb amputations at proximal levels. The remaining 45% (n = 148) were minor amputations, encompassing amputation at the hand or wrist, foot or ankle, and fingers and toes.

Causes of amputations were coded according to the CID-10^8^ (Table 2), and it was found that 80% (n = 244) were related to the conditions listed in chapter I, followed by chapter IX, with 10.4% (n = 32), and chapter XVIII, with 4.2% (n = 13) of amputations. The other chapters of the CID-10 were related to lower percentages.

In this study, 96.4% (n = 264) of amputations were performed on patients admitted through the hospital’s emergency sector. The majority of amputees recovered and were discharged (69.7%; n = 191), but a considerable proportion died (21.9%; n = 60) (Table 3).

**Table 3:** Distribution of patients according to the characteristics of admission. Recife, PE, Brazil, 2019.

**Characteristics of admission**	**n**	**%**
Type of care, n = 274		
Elective	10	3.6
Urgent	264	96.4
Cause of exit, n = 274		
Discharge	191	69.7
Death	60	21.9
Transfer	23	8.4
Length of hospital stay, in days (median; minimum-maximum), n = 274	21	1-299
1 to 10 days	76	27.7
11 to 25 days	88	32.1
26 to 50 days	71	25.9
51 to 100 days	31	11.3
More than 100 days	8	2.9

Deaths were predominantly related to non-traumatic amputations and LL amputations. Patients who underwent LL amputation and then died were predominately elderly women in the 60 to 90 years age group (76%; n = 44), females (55%; n = 32), and had single amputations (91%; n = 53). Furthermore, a large proportion of these deaths occurred within one month (30 days) of amputation, among both LL amputations (58%; n = 34) and UL amputations (100%; n = 2) (Table 4).

**Table 4:** Characteristics of deaths by level of amputations. Recife, PE, Brazil, 2019.

	**Deaths**			
	**LL** **n = 58**		**UL** **n = 2**	
**Sex**	**n**	**%**	**n**	**%**
Female	32	55%	1	50%
Male	26	45%	1	50%
**Age**				
13-59 years	10	17%	2	100%
60- 90 years	44	76%	-	-
More than 90 years	4	7%	-	-
**Type of amputation**				
Traumatic	3	5%	1	50%
Non-traumatic	55	95%	1	50%
**Number of amputations**				
One	53	91%	2	100%
Two	4	7%	-	-
Three	1	2%	-	-
**Cause of amputation, n = 64/n = 2**				
I Certain infectious and parasitic diseases	53	83%	1	50%
II Neoplasms [tumors]	-	-	1	50%
IX Diseases of the circulatory system	3	5%	-	-
XII Diseases of the skin and subcutaneous tissue	2	3%	-	-
XVIII Symptoms, signs and abnormal clinical and laboratory findings, not elsewhere classified	5	8%	-	-
XIX Injury, poisoning and certain other consequences of external causes	1	2%	-	-
**Type of care**				
Elective	2	3%	-	-
Urgent	56	97%	2	100%
**Length of hospital stay (days)**				
1 to 10 days	21	36%	2	100%
11 to 25 days	13	22%		-
26 to 50 days	14	24%		-
51 to 100 days	8	14%		-
More than 100 days	2	3%		-

UL: upper limbs, including hand and wrist; LL: lower limbs, including amputations at toes, foot, and ankle.

With regard to length of hospital stay, the majority of amputation patients remained from 11 to 25 days (32.1%; n = 88) in hospital, although some were in hospital for more than 100 days (Table 3).

## DISCUSSION

It was found that the majority of patients who underwent amputations were elderly, which is a similar result to those of previous studies of amputation, particularly non-traumatic amputations.^3^^,^ 9 ^,^ 10

In this study, the majority of amputated patients were male, which may be because men are less likely to seek health care, are more exposed to risk situations, and are more likely to neglect their health care.^11^

A study conducted in the United States showed that men are more likely to undergo LL amputations and suggested that women have higher mortality rates associated with these amputations, both of which were characteristics also observed in this study. The same US study also revealed that men are more likely to have diabetic foot, to smoke cigarettes, and to have peripheral neuropathy, and had almost double the likelihood of neuropathy with insensitivity than women. Hormonal factors and men’s height were mentioned as possible factors in these findings.^12^

The majority of patients had a single amputation, but in the majority of cases it involved a large part of the limb, indicating that the limb was in a severely compromised state, in an irreversible condition. This raises the question of whether these people are seeking healthcare late or if the healthcare system is failing to avoid deterioration in their clinical status in time to save the greatest possible proportion of the limb.

With regard to number of amputations, one patient underwent three amputations, starting with the most distal level possible. This finding could be clarified considering that the decision to amputate should always be taken in context and considering all other possible measures, to guarantee better quality of life and limb functionality. Therefore, the level of amputation is considered before the procedure with the objective of preserving as much of the limb as possible and ensuring a good healing process, adequate skin coverage, and preserved local sensitivity. However, this is not always possible and it can be necessary to reoperate and amputate at a more proximal level, which is what happened with some of the patients in this study.^2^

In this regard, a study by Thorud et al.^13^ reported a reamputation rate estimated at 28.37% of patients who underwent transmetatarsal amputations, whereas in this study the reamputation rate was 17.1%. While this is a lower rate than in the study mentioned, it should be emphasized that the majority of the patients in this study had LL amputations at proximal levels, defined as major amputations, because the limb was highly compromised, so reamputation was not possible.

Along the same lines, another study listed the factors associated with readmissions and reamputations, such as elective admission, peripheral arterial disease, chronic renal failure, infection, and ischemia, and observed that patient comorbidities had a direct influence on the risk of patient readmission, the risk of reamputation, and the risk of mortality.^14^

The higher rate of LL amputations than UL amputations can also be found in other studies on the subject.^1^^,^ 2 ^,^ 10 ^,^ 15 Amputations of upper limbs, and particularly major ones, are most often performed on young adults, males, of working age, and tend to be caused by blunt trauma caused by occupational accidents, traffic accidents, and projectile wounds.^16^ In contrast, LL amputations are more common among older people and are mostly related to non-traumatic causes, such as DM and vascular diseases.^3^^,^ 10 ^,^ 17 ^,^ 18 In this study, upper limb amputations accounted for a much lower percentage than LL amputations, involved a younger age group, and the majority were caused by traumatic events.

In Brazil, data from the National Health Service database show an upward trend in the number of amputations, increasing from approximately 42 thousand amputations in 2010 to more than 55 thousand in 2017. With regard to type of amputation, the system shows a prevalence of LL amputations over the years, which is a loss that involves high costs, prolonged hospital stays, need for rehabilitation, and reduced quality of life.^6^

With regard to this issue, there are studies indicating that the incidence of major amputations may be more related to vascular causes, because of atherosclerotic disease and DM.^3^ A study by Borges^15^ with patients with chronic LL ischemia indicated that the most common risk factors for major amputations observed were chronic kidney disease, coronary artery disease, uncontrolled DM, arterial aneurysms, previous amputations, and lack of primary care.

According to estimates by the Brazilian Society for Angiology and Vascular Surgery (SBACV - Sociedade Brasileira de Angiologia e de Cirurgia Vascular), diabetic patients are 15 to 30 times more likely to undergo an LL amputation than non-diabetic patients and account for 80% of non-traumatic amputations, with an incidence of 50-90/10,000 patients with DM per year. The SBACV also reports that 25% of diabetic patients will have an LL ulcer at some point in their lives, that 50% of these ulcers will become infected, and that amputation will be the outcome in 20%.^19^

Thought must be given to how to avoid people arriving at the point at which they have to undergo an amputation, despite having access to a healthcare system with several different levels of care and a healthcare network intended to provide integrated care.

It was notable that gangrene was listed as the cause of the majority of amputations. Gangrene is in chapter I of the CID-10^8^, but it was not possible to link it to diseases/causes/comorbidities, because there was no additional information recorded on patient records. Gangrene was considered the cause of amputations related to clinical conditions and also of traumatic amputations and wet and gas gangrenes were the most prevalent forms.

It is important to point out that gangrene is very common among patients who have DM; 100 times more common among DM patients than in the general population, because DM causes hypercholesterolemia, which increases the risk of atherosclerosis and, consequently, of gangrene of the extremities. Notwithstanding, traumatic and surgical wounds can also cause gangrene, primarily gas gangrene, because they increase the risk of bacterial contamination.^20^

The majority of amputations involved LL and, as explained above, limbs were highly compromised, there was a higher number of urgent treatments. On this issue, a study by Prin et al.^21^ showed the extent to which the proportions of urgent and elective surgeries are disproportionate in different parts of the world, such as sub-Saharan Africa, the United States, and Europe. Developing countries have many more difficulties with access to healthcare, whereas in high income nations care is better and the numbers of emergency surgeries are much lower.

The high mortality rates associated with emergency surgeries, compared with elective surgeries show how the existence of an organized healthcare system has a great influence on postoperative outcomes. It is important to identify patients at greater risk and provide early treatment of complications. However, certain factors interfere with this process, such as large numbers of patients, a nursing team that is unable to deal with the demand it is subjected to, communication failures, and an absence of care escalation.^22^

This study found that a considerable number of patients were discharged after the procedure; but, as observed in a study by Rolim et al.^23^ the mortality rate was very high, and particularly so after LL amputation. In that study, the overall mortality rate increased at 30 days, 90 days, 365 days, and 5 years after amputation, at 12%, 23%, 33%, and 59%, respectively. The study authors pointed out that the higher the level of amputation, the higher the mortality rate. They also linked their results to the ongoing aging process, to the increased number of comorbidities, and to commitment to recovering the limb.

With regard to length of hospital stay, this varies according to the type of amputation (traumatic or non-traumatic), patient age, postoperative complications, and hospital logistics, among other factors. In a study by Senefonte et al.,^5^ mean length of hospital stay was 23.7±7.8 days, contrasting with a study by Bortoletto et al.,^24^ in which length of hospital stay ranged from 3 to 50 days. Nevertheless, in studies such as one by Oliveira et al.,^25^ the length of hospital stays of amputated patients ranged from 7 to 157 days, similar to in this study.

The data reported in this study are alarming, particularly if it is considered that many of these amputations could have been avoided if healthcare actions had been scaled up long before development of the health problem or at least sufficiently to avoid major and irreversible compromise. It is therefore clear that integrated care may seem close on paper, but is still a long way off in reality.

These data emphasize the importance of improved vascular screening and early detection, improved vascular care, and an optimized revascularization policy in Brazil, if we are to have quality healthcare in practice. This study has afforded an overview of the panorama of amputations in the region and can serve as a foundation for improvement actions targeting the population, thereby facilitating a reduction in the number of amputations and improving people’s quality of life.

This study revealed and analyzed a great deal of information that is relevant to the institution where it was conducted and also for promotion of knowledge about amputations in general. Notwithstanding, it also has certain limitations, including missing data and a lack of additional variables that could have been studied, such as costs, educational level, presence of chronic diseases, time since diagnosis, and history of prior amputations. A lack of a better system for organizing data on amputations in the hospital records also caused problems for the study, leading to losses, and the unavailability of notification services meant there was no way of obtaining information on survival after discharge, while logistical factors prevented dedicating a longer period of time to research and data collection.

In conclusion, the study revealed that, among the 328 procedures performed, the number of LL amputations was highest, that amputees were predominantly in the 60 to 90 years age group and male. Major amputations, patients who underwent a single amputation, non traumatic amputations, and emergency procedures all predominated. The most frequent hospital stay period was 11-25 days, the majority of patients were discharged from hospital, but many died in hospital, particular elderly patients, and females, who underwent LL amputations. Gangrene was the number one cause of amputation.

Taking into consideration the facts mentioned, this study hopes to raise awareness about the incidence of amputations in Brazil, considering the effect that they can have on amputees. By focusing attention and care on prevention of amputations, both sides will gain. Primary care should be prepared for screening and early detection of people who have health problems involving a high likelihood of a future amputation, in addition to providing a referral path for patients to access the care needed, primarily vascular care.
