# Supplementary material for: Characterization of amputees at a large hospital in Recife, PE, Brazil
Source: J Vasc Bras. 2019 Oct 23;18:e20190064. doi: 10.1590/1677-5449.190064 (PMC6822958; doi:10.1590/1677-5449.190064)
Supplement: Translation

# Translation: Caracterização das pessoas amputadas de um hospital de grande porte em Recife (PE, Brasil)

**Authors:** Ylkiany Pereira de Souza, Ana Célia Oliveira dos Santos, Luciana Camelo de Albuquerque

**Affiliations:** 1 Universidade Federal de Pernambuco – UFPE, Programa de Pós-graduação em Gerontologia, Recife, PE, Brasil.; 2 Universidade de Pernambuco – UPE, Instituto de Ciências Biológicas – ICB, Recife, PE, Brasil.; 3 Prefeitura da Cidade de Recife, Distrito Sanitário 2, Recife, PE, Brasil.

**Keywords:** amputação, desarticulação, membros superiores, membros inferiores

## Abstract

**Contexto:**

A amputação de membros pode ser definida como um procedimento que consiste em separar do corpo um membro ou segmento dele.

**Objetivos:**

Descrever o perfil dos procedimentos de amputações de membros realizados em um hospital estadual de grande porte.

**Métodos:**

Estudo transversal do tipo descritivo e retrospectivo realizado em um hospital de grande porte da cidade de Recife (PE). Os dados foram coletados em registros de pacientes que foram submetidos à amputação no ano de 2017. Foram incluídos pacientes que tiveram seu membro amputado em 2017 e excluídos aqueles com informações ilegíveis ou incompletas.

**Resultados:**

Foram realizados 328 procedimentos em 274 pacientes, a maioria do gênero masculino (57,7%). Houve predominância de amputações em membros inferiores (64,2%), de causa não traumática (86,5%) e atendimentos de urgência (96,4%). A maioria dos pacientes submetidos a amputações ficaram internados por um período de 11 a 25 dias (32,1%). Este estudo mostrou que a maioria das pessoas amputadas recebeu alta (69,7%) e uma parte foi a óbito, sendo os óbitos, no caso das amputações de membros inferiores, principalmente de pessoas idosas na faixa etária de 60 a 90 anos (76%), do sexo feminino (55%), e submetidas a uma amputação (91%).

**Conclusões:**

Os dados apresentados neste estudo são alarmantes, principalmente ao se considerar que muitas das amputações poderiam ter sido evitadas, visto que foram decorrentes de complicações de doenças cuja prevenção e controle podem ser realizados em outros níveis de complexidade do sistema e com custo relativamente baixo.

## INTRODUÇÃO

A amputação de membros é um grande problema de saúde pública, sendo responsável por altas taxas de morbimortalidade, e consiste na perda de um membro ou segmento dele, causando impacto no aspecto psicossocial do indivíduo e representando também um fator oneroso para a saúde. Geralmente é realizada como último recurso, na busca pelo reestabelecimento da saúde do indivíduo, quando todas as outras opções já foram utilizadas ou são inviáveis, seja por conta de isquemias irreversíveis ou trauma com destruição profunda do membro afetado^1^^-^ 3.

Entre as causas que levam a uma amputação estão as doenças infecciosas e parasitárias, doenças do aparelho circulatório, diabetes melito (DM), gangrena, doenças do sistema osteomuscular e do tecido conjuntivo, neoplasias, causas externas, doenças de pele e malformações congênitas^2^. Assim, as cirurgias de amputação podem ser realizadas em caráter de urgência ou de forma eletiva^1^.

Segundo os poucos estudos sobre a epidemiologia das amputações, estima-se que a incidência mundial de amputação de membros seja de aproximadamente 1 milhão de pessoas por ano^4^^,^ 5. No ano de 2018, foram registradas no Brasil mais de 59 mil amputações, das quais 2.694 ocorreram no estado de Pernambuco, colocando o estado em oitavo lugar em número de amputações^6^.

Os membros inferiores (MMII) geralmente são os mais acometidos em comparação aos membros superiores (MMSS). Segundo dados do Ministério da Saúde (MS), em 2011 a amputação de MMII foi responsável por cerca de 94% de todas as amputações realizadas^2^.

O objetivo deste estudo foi descrever o perfil dos procedimentos de amputação de membros realizados em um hospital estadual de grande porte. Os resultados poderão subsidiar os gestores na tomada de decisão para oferta de políticas públicas que possam reduzir a quantidade de amputações.

## MÉTODO

Trata-se de um estudo transversal do tipo descritivo e retrospectivo realizado em um hospital de grande porte localizado na cidade de Recife (PE) com o intuito de analisar dados hospitalares de pacientes que foram submetidos a amputação de membros no período de janeiro de 2017 a dezembro de 2017.

A coleta de dados foi realizada entre os meses de maio a setembro de 2018, por meio de um formulário, aplicado por um único examinador, que continha as seguintes variáveis: sexo, idade, tipo de amputação, procedimento realizado, caráter de atendimento, número de amputações, causa da amputação, procedência, motivo da saída do hospital e tempo de internação.

Para confirmar a quantidade de amputações realizadas no período e obter informações sobre todas as variáveis previstas no instrumento, o pesquisador aplicou o formulário em três fontes de dados: 1) registro do centro cirúrgico, utilizando os livros de registro das cirurgias; 2) documento de encaminhamento para o necrotério; e 3) espelhos da autorização de internação hospitalar (AIH) disponíveis no setor do faturamento, por meio do Programa de Apoio a Entrada de Dados das AIH (SISAIH01).

Convém salientar que, como foi utilizado o SISAIH01 para a obtenção dos espelhos da AIH, não foi possível estabelecer com precisão o nível de amputação realizado, ou seja, se foi transtibial, transfemural, etc., porque o sistema engloba vários níveis em uma mesma região. Assim, os exemplos acima citados estão contidos dentro da categoria amputação/desarticulação de MMII.

No centro cirúrgico, foram coletados 330 registros de pessoas submetidas a amputações de membros, e no setor do necrotério foram encontrados 200 registros. Percebeu-se então que muitos registros do bloco cirúrgico não constavam no necrotério e vice-versa, sendo que todos os membros amputados são encaminhados para o necrotério com o documento de encaminhamento. Assim, dos 330 registros coletados no bloco cirúrgico, 151 coincidiram com as informações do necrotério, e foram encontrados 49 registros a mais. No setor do faturamento, foram utilizados os 330 registros do centro cirúrgico e mais os 49 do necrotério que não constavam no centro cirúrgico, totalizando 379 registros para pesquisa. Entretanto, alguns foram excluídos durante a análise dos espelhos, devido aos seguintes motivos: espelhos incompletos, registros não coincidiam com o nome, espelhos que não condiziam com amputação, registros repetidos, e registros não encontrados. Dessa forma, contabilizou-se no final 274 registros coletados (Figura 1), com uma perda de 105.

**Figura 1:** Diagrama de fluxo das fases da coleta de dados, mostrando o número de registros identificados, selecionados e incluídos na pesquisa. AIH = autorização de internação hospitalar.

Os dados coletados foram armazenados no Microsoft Excel® para análise descritiva dos dados, em que foram obtidas frequências absoluta (n) e relativa (%) com respeito às variáveis de interesse categóricas/qualitativas e, no caso de variáveis quantitativas, estas foram apresentadas sob a forma de média (± desvio padrão). A pesquisa atendeu a todos os aspectos éticos da pesquisa com seres humanos, conforme a Resolução 466/2012^7^, e foi submetida a um Comitê de Ética em Pesquisa, por meio da Plataforma Brasil, sendo aprovado sob o parecer n° 2.622.379.

## RESULTADOS

Foram realizados 328 procedimentos de amputação em 274 pacientes com idades que variaram de 13 anos a > 90 anos, com média de 63 anos (desvio padrão ±16). A maior frequência de pacientes amputados foi observada no grupo etário de 60 a 90 anos (60,2%; n =165), e a maioria das pessoas amputadas (57,7%; n = 158) era do sexo masculino (Tabela 1).

**Tabela 1:** Distribuição dos pacientes de acordo com as variáveis sociodemográficas do estudo. Recife (PE), Brasil, 2019.

**Características gerais**	**n**	**%**
Idade (média ± desvio padrão), n = 274	63±16	
13 a 59 anos	100	36,5
60 a 90 anos	165	60,2
Mais de 90 anos	9	3,3
Sexo, n = 274		
Masculino	158	57,7
Feminino	116	42,3

As amputações não traumáticas foram as mais realizadas, alcançando um percentual de 86,5% (n = 237). Quando analisada a quantidade de amputações, identificou-se que a grande maioria dos pacientes (83,2%; n = 228) foi submetida a uma amputação. No que se refere ao procedimento realizado, a amputação/desarticulação de MMII foi a mais prevalente (53,6%; n = 176), seguida de amputação/desarticulação de dedo (23,4%; n = 77) (Tabela 2).

**Tabela 2:** Distribuição dos pacientes de acordo com as características das amputações do estudo. Recife (PE), Brasil, 2019.

**Características gerais**	**n**	**%**
Tipo de amputação, n = 274		
Traumática	37	13,5
Não traumática	237	86,5
Número de amputações realizadas, n = 274		
Uma	228	83,2
Duas	38	13,8
Três	8	2,9
Procedimento de amputação, n = 328		
Amputação/desarticulação de MMSS	4	1,2
Amputação/desarticulação de mão e punho	2	0,6
Amputação/desarticulação de MMII	176	53,6
Amputação/desarticulação de pé e tarso	69	21
Amputação/desarticulação de dedo	77	23,4
Causa da amputação, n = 305		
I Algumas doenças infecciosas e parasitárias	244	80
II Neoplasmas [tumores]	2	0,6
IV Doenças endócrinas, nutricionais e metabólicas	2	0,6
IX Doenças do aparelho circulatório	32	10,4
XII Doenças da pele e do tecido subcutâneo	2	0,6
XIII Doenças do sistema osteomuscular e do tecido conjuntivo	1	0,3
XVIII Sintomas, sinais e achados anormais de exames clínicos e de laboratório, não classificados em outra parte	13	4,2
XIX Lesões, envenenamentos e algumas outras consequências de causas externas	8	2,6
XXI Fatores que influenciam o estado de saúde e o contato com os serviços de saúde	1	0,3

MMSS: membros superiores; MMII: membros inferiores.

Com relação à quantidade de amputações maiores (aquelas realizadas mais a nível proximal) e menores (realizadas a níveis distais), observou-se que 54,8% (n = 180) das amputações foram amputações maiores, que correspondem às amputações de MMII – sigla utilizada pelo Sistema de Informações Hospitalares do Sistema Único de Saúde (SIH-SUS) para amputações em MMII a níveis proximais – mais as de MMSS – sigla utilizada pelo SIH-SUS para amputações em membros superiores a níveis proximais –, e 45% (n = 148) foram amputações menores resultantes da amputação de mão e punho, pé e tarso, ou dedo.

As causas das amputações foram agrupadas de acordo com o CID-10^8^ (Tabela 2), observando-se que 80% (n = 244) foram relacionadas a afecções presentes no capítulo I, seguida do capitulo IX, com 10,4% (n = 32), e do capítulo XVIII, responsável por 4,2% (n = 13) das amputações. Os demais capítulos do CID-10 apresentaram menores percentuais.

Neste estudo, 96,4% (n = 264) das amputações foram realizadas em pacientes admitidos na urgência do hospital. Entre os amputados, a maioria obteve melhora com alta (69,7%; n = 191), e uma parcela considerável foi a óbito (21,9%; n = 60) (Tabela 3).

**Tabela 3:** Distribuição dos pacientes de acordo com as características da internação do estudo. Recife (PE), Brasil, 2019.

Características da internação	n	%
Caráter atendimento, n = 274		
Eletiva	10	3,6
Urgência	264	96,4
Motivo da saída, n = 274		
Alta	191	69,7
Óbito	60	21,9
Transferência	23	8,4
Tempo de internação, em dias (mediana; mínimo-máximo), n = 274	21	1-299
1 a 10 dias	76	27,7
11 a 25 dias	88	32,1
26 a 50 dias	71	25,9
51 a 100 dias	31	11,3
Mais de 100 dias	8	2,9

Os óbitos foram predominantemente relacionados a amputações não traumáticas e em MMII. Entre os pacientes que sofreram amputação de MMII e vieram a óbito, predominaram pessoas idosas na faixa etária de 60 a 90 anos (76%; n = 44), do sexo feminino (55%; n = 32), e submetidas a uma amputação (91%; n = 53). Além disso, grande parte dos óbitos ocorreu dentro de um mês (30 dias) após a amputação, tanto em amputações em membros inferiores (58%; n = 34) como superiores (100%; n = 2) (Tabela 4).

**Tabela 4:** Caracterização dos óbitos de acordo com o nível das amputações do estudo. Recife (PE), Brasil, 2019.

	**Óbitos**			
	**MMII** **n = 58**		**MMSS** **n = 2**	
**Sexo**	**N**	**%**	**n**	**%**
Feminino	32	55%	1	50%
Masculino	26	45%	1	50%
**Idade**				
13-59 anos	10	17%	2	100%
60-90 anos	44	76%	-	-
Mais de 90 anos	4	7%	-	-
**Tipo de amputação**				
Traumática	3	5%	1	50%
Não traumática	55	95%	1	50%
**Número de amputações**				
Uma	53	91%	2	100%
Duas	4	7%	-	-
Três	1	2%	-	-
**Causa da amputação, n = 64/n = 2**				
I Algumas doenças infecciosas e parasitárias	53	83%	1	50%
II Neoplasmas [tumores]	-	-	1	50%
IX Doenças do aparelho circulatório	3	5%	-	-
XII Doenças da pele e do tecido subcutâneo	2	3%	-	-
XVIII Sintomas, sinais e achados anormais de exames clínicos e de laboratório, não classificados em outra parte	5	8%	-	-
XIX Lesões, envenenamentos e algumas outras consequências de causas externas	1	2%	-	-
**Caráter do atendimento**				
Eletiva	2	3%	-	-
Urgência	56	97%	2	100%
**Tempo de internação (dias)**				
1 a 10 dias	21	36%	2	100%
11 a 25 dias	13	22%	-	-
26 a 50 dias	14	24%	-	-
51 a 100 dias	8	14%	-	-
Mais de 100 dias	2	3%	-	-

MMSS: membros superiores, incluindo mão e punho; MMII: membros inferiores, incluindo as amputações de dedo e pé e tarso.

Quanto ao tempo de internação, a maioria dos pacientes submetidos a amputações ficaram internados de 11 a 25 dias (32,1%; n = 88), e alguns ultrapassaram 100 dias de internação (Tabela 3).

## DISCUSSÃO

Percebeu-se que a maioria dos pacientes submetidos a amputação eram idosos, resultado semelhante ao de estudos anteriores sobre amputação, principalmente amputação não traumática^3^^,^ 9 ^,^ 10.

Neste estudo, a maioria dos pacientes submetidos a amputações eram do sexo masculino, o que pode ser explicado pelo fato de os homens serem mais relutantes em procurar assistência à saúde, ficarem mais expostos a situações de risco e negligenciarem mais seus cuidados com a saúde^11^.

Um estudo estadunidense mostrou que os homens são mais propensos a se submeterem a amputações em MMII e sugere que as mulheres têm maiores taxas de mortalidade associadas com essas amputações, ambas caracteristicas encontradas neste estudo. A pesquisa acima também apontou que os homens são mais propensos a terem pé diabético, fazerem uso do cigarro e apresentarem neuropatia periférica, tendo quase o dobro de chances de apresentar neuropatia insensível do que as mulheres. Fatores hormonais e a altura dos homens são citados como possíveis fatores influenciadores^12^.

A maioria dos pacientes foi submetida a uma amputação, porém na maioria das vezes envolvendo grande parte do membro, um indicativo de que o estado do membro estava altamente afetado, com irreversibilidade do caso. Surge então o questionamento se são as pessoas que estão procurando o serviço de saúde tardiamente ou se o sistema de saúde não está conseguindo evitar a piora do quadro clínico a tempo de salvar a maior parte do membro possível.

Em relação ao número de amputações, um mesmo paciente passou por três amputações, começando pela retirada do menor nível possível. Esse dado pode ser elucidado tendo em vista que a decisão pela amputação deve sempre ser trabalhada em cima de um contexto e considerar todas as outras medidas possíveis, para garantir uma melhor qualidade de vida e funcionalidade dos membros. Assim, o nível de amputação é considerado antes do procedimento no intuito de preservar o máximo de membro possível e garantir um bom processo de cicatrização, uma cobertura adequada da pele e a preservação da sensibilidade no local – no entanto, isso nem sempre é possível, sendo necessário aumentar o nível da amputação, que foi o que ocorreu com alguns dos pacientes desse estudo^2^.

A esse respeito, no estudo de Thorud et al.^13^ a taxa de reamputação foi estimada em 28,37% dos pacientes submetidos a amputações transmetatarsais, enquanto que neste estudo a taxa de reamputação foi de 17,1%. Embora essa taxa seja inferior à do estudo citado, ressalta-se que no presente estudo a maioria dos pacientes foi submetido a amputação de MMII a níveis proximais, considerada uma amputação maior, pois o membro estava altamente comprometido, não havendo chances de reamputação.

Nessa mesma linha, outro estudo apontou fatores associados a readimissões e reamputações de pacientes, tais como internação eletiva, doença arterial periférica, insuficiência renal crônica, infecção e isquemia, e observou que a comorbidade do paciente influencia diretamente o risco de readmissão do paciente, bem como o risco de reamputação e mortalidade^14^.

A maior taxa de amputações em MMII em relação a membros superiores pode ser observada nos diferentes estudos sobre o tema^1^^,^ 2 ^,^ 10 ^,^ 15. As amputações em membros superiores, principalmente as de maior extensão, atingem com mais frequência adultos jovens, do sexo masculino e em idade produtiva, e costumam ser decorrentes de trauma contuso causado por acidentes de trabalho, automobilísticos e com projéteis^16^. Diferentemente, as amputações em MMII atingem mais idosos e estão relacionadas em grande parte a causas não traumáticas, como DM e doenças vasculares^3^^,^ 10 ^,^ 17 ^,^ 18. Neste estudo, observou-se que amputações em membros superiores representavam um percentual bem menor em relação às de MMII, além de atingirem uma faixa etária mais jovem e serem decorrente majoritariamente de eventos traumáticos.

No Brasil, dados do SIH-SUS mostram uma tendência crescente no número de amputações, aumentando de aproximadamente 42 mil amputações em 2010 para mais de 55 mil em 2017. Quanto ao nível de amputação, o sistema mostrou prevalência das amputações de MMII ao longo dos anos, uma perda que acarreta altos custos, tempo de internação prolongado, necessidade de reabilitação, diminuição na qualidade de vida, entre outros^6^.

Sobre esse assunto, existem estudos indicando que a incidência de amputações maiores pode estar mais relacionada a causas vasculares, por conta da doença aterosclerótica e DM^3^. Estudo de Borges^15^ em pacientes com isquemia crônica dos MMII apontou que os fatores de risco para amputações maiores mais encontrados foram doença renal crônica, doença arterial coronariana, DM descontrolado, aneurisma arterial, amputação prévia e falta de atendimento na atenção básica.

Segundo estimativas da Sociedade Brasileira de Angiologia e de Cirurgia Vascular (SBACV), pacientes diabéticos têm 15 a 30 vezes mais chances de sofrerem amputação em MMII em comparação a pacientes não diabéticos e são responsáveis por 80% das amputações não traumáticas, com uma incidência de 50-90/10.000 pacientes com DM por ano. A SBACV também salienta que 25% dos pacientes diabéticos terão em algum momento da vida úlcera em MMII, sendo que 50% dessas úlceras se tornarão infectadas e 20% terão como desfecho a amputação^19^.

Deve-se pensar em como evitar que uma pessoa, mesmo inserida em um sistema com vários níveis de complexidade de assistência e com redes de atenção a saúde para garantir um cuidado integral, tenha que no final ser submetida a um procedimento de amputação de um membro.

Destaca-se que a gangrena foi considerada como a causa da maioria das amputações, estando presente no capítulo I da CID-10^8^. Entretanto, não foi possível relacionar doença/causa/comorbidades, porque não havia o registro de informações dos prontuários. No que se refere à gangrena, esta foi considerada como causa tanto nas amputações relacionadas a afecções clínicas como nas amputações traumáticas, sendo as gangrenas úmida e gasosa as mais prevalentes.

É importante destacar que a gangrena é muito frequente em pacientes que apresentam DM, sendo 100 vezes mais comum nesses pacientes do que na população em geral, pois a DM induz a hipercolesterolemia, a qual aumenta o risco de aterosclerose e consequentemente de gangrena nas extremidades. Todavia, as lesões traumáticas e cirúrgicas também podem causar gangrena, principalmente a gangrena gasosa, por aumentarem o risco de contaminação bacteriana^20^.

Considerando que a maioria das amputações foi de MMII e, como elucidado anteriormente, com o membro em alto grau de comprometimento, houve uma maior quantidade de atendimentos de urgência. Sobre isso, estudo realizado por Prin et al.^21^ mostrou o quão desproporcionais são os números de cirurgias de urgência comparadas com as cirurgias eletivas em diferentes regiões do mundo, tais como África subsaariana, Estados Unidos e países europeus. Os países em desenvolvimento têm muito mais dificuldades de acesso à assistência a saúde, enquanto em nações de alta renda essa assistência é mais adequada e o número de cirurgias de emergência bem menor.

As altas taxas de mortalidade associadas às cirurgias de emergências, em comparação às cirurgias eletivas, mostram como a presença de um sistema de saúde organizado tem grande influência nos desfechos pós-operatórios. Para isso, é importante conhecer os pacientes de maior risco e realizar um tratamento precoce das complicações. Porém, alguns fatores acabam dificultando esse processo, como grande número de pacientes, presença de uma equipe de enfermagem que não consegue atender toda a demanda, falhas na comunicação e ausência de escalonamento de risco^22^.

Este estudo mostrou considerável número de pacientes que receberam alta após o procedimento; porém, como refere o estudo de Rolim et al.^23^ a taxa de mortalidade, principalmente após amputação de MMII, é muito alta. No estudo acima, a taxa de mortalidade global aumentou aos 30, 90, 365 dias e aos 5 anos após a amputação, chegando a 12%, 23%%, 33% e 59%, respectivamente. Os autores do estudo destacaram que, quanto maior o nível da amputação, maior a taxa de mortalidade; além disso, associaram seus resultados ao crescente processo de envelhecimento, ao aumento do número de comorbidades e ao comprometimento na recuperação do membro.

Quanto ao tempo de internação, este varia de acordo com o tipo de amputação (traumática e não traumática), idade dos pacientes, complicações pós-cirúrgicas, logística hospitalar, entre outros fatores. No estudo de Senefonte et al.^5^, a média do tempo de internação foi de 23,7±7,8 dias, contrastando com o estudo de Bortoletto et al.^24^, no qual o tempo de internação variou de 3 a 50 dias. Todavia, em estudos como o de Oliveira et al.^25^, o tempo de hospitalização dos pacientes que amputaram variou de 7 a 157 dias, resultado semelhante ao do presente estudo.

Os dados apresentados nesta pesquisa são alarmantes, principalmente ao se considerar que muitas das amputações poderiam ter sido evitadas se ações de saúde fossem fortalecidas muito antes do desenvolvimento do problema de saúde ou de modo a evitar um grande comprometimento irreversível. Percebe-se, portanto, que a integralidade no cuidar está perto no papel, mas ainda longe da realidade.

Assim, destaca-se a importância do rastreio, detecção precoce, cuidados vasculares melhorados e uma política de revascularização otimizada no país, para que efetivamente tenhamos uma saúde de qualidade. Este estudo possibilitou uma visão sobre o panorama das amputações na região e pode subsidiar ações de melhorias para a população, favorecendo assim uma possível redução no número de amputações e melhorando a qualidade de vida das pessoas.

O estudo possibilitou o conhecimento e a análise de muitas informações relevantes tanto para a instituição onde foi realizada a pesquisa como para a promoção do conhecimento sobre amputações. Porém, apresentou algumas limitações, incluindo insuficiência de dados e ausência de mais variáveis a serem estudadas, tais como custos, escolaridade, presença de doenças crônicas, tempo de diagnostico e histórico de amputação prévia. A falta de melhor organização dos dados sobre amputação nos registros do hospital também dificultou a pesquisa, causando perdas, bem como a indisponibilidade de serviços de notificação, não sendo possível ter conhecimento sobre a sobrevida após a alta, e uma logística que impossibilitou maior tempo para a pesquisa e para a coleta.

Em conclusão, o estudo revelou que, dos 328 procedimentos realizados, o número de amputações em MMII foi o mais elevado, estando as amputações presentes, sobretudo, no grupo etário de 60 a 90 anos e em homens. Houve predominância de amputações maiores, pacientes submetidos a uma única amputação, amputações não traumáticas e atendimentos de urgência. O tempo da internação mais frequente foi o de 11-25 dias, a maioria dos pacientes recebeu alta hospitalar, e uma parte foi a óbito, sobretudo pacientes idosos, do gênero feminino, e submetidos a amputações em MMII. A formação de gangrena foi o principal motivo que levou à amputação.

Levando-se em consideração os fatos mencionados, o estudo buscou alertar sobre a incidência de amputações no país, considerando como isso pode afetar o indivíduo amputado. Ao focar a atenção e o cuidado na prevenção das amputações, os ganhos serão para as ambas as partes. A atenção básica deve ser preparada para o rastreio e a detecção precoce de indivíduos que apresentem problemas de saúde com grandes chances de uma futura amputação, além de proporcionar ao paciente uma via de referência para receber os cuidados adequados, principalmente vasculares.
